# What Air Shall We Breathe at Night?

**Published:** 1881-06

**Authors:** 


### WHAT AIR SHALL WE BREATHE AT NIGHT?

Many years ago, Florence Nightingale assaulted the popular superstition against breathing night air with the unanswerable question, What other air can you breathe at night? Dr. Oswald, in the last number of the *Popular Science Monthly*, enters upon the assault against this superstition, which survives every attack, upon no other ascertainable ground than the less reason there is for a superstition the harder it is to kill it.

"Before we can hope," he says, "to get rid of the consumption, with any chance of success, we have to get rid of the night-air superstition. It is probably the most prolific single cause of impaired health, even among the civilized nations of our enlightened age, though its absurdity rivals the grossest delusions of the witchcraft era. The subject of holy reason to hearsays could hardly go further.

"Beware of the night-wind; be sure and close your windows after dark! In other words, beware of God's free air; be sure and infect your lungs with the stagnant, azotized, and offensive atmosphere of your bed-room. In other words, beware of the rock spring; stick to sewerage. Is night air injurious? Is there a single tenable pretext for such an idea? Since the day of creation that air has been breathed with impunity by millions of different animals—tender, delicate creatures, some of them—fawns, lambs, and young birds. The moist night air of the tropical forest is breathed with impunity by our next relatives, the anthropoid apes—the same that soon perish with consumption in the close, though generally well-warmed atmosphere of our northern menageries. Thousands of soldiers, hunters and lumbermen sleep every night in tents and open sheds without the least injurious consequences ; men in the last stage of consumption have recovered by adopting a semi-savage mode of life and camping out doors in all but the stormiest nights. Is it the draught you fear, or the contrast of temperature ? Blacksmiths and railroad conductors seem to thrive under such influences. Draught ? Have you never seen boys skating in the teeth of a snow-storm at the rate of fifteen miles an hour ? 'They counteract the effect of the cold air by vigorous exercise.' Is there no other way of keeping warm ? Does the north wind damage the fine lady sitting motionless in her sleigh, or the pilot and helmsman of a storm-tossed vessel ? It cannot be the inclemency of the open air, for, even the sweltering summer nights, the sweet south wind, blessed by all creatures that draw the breath of life, brings no relief to the victim of aerophcbia. There is no doubt that families who have freed themselves from the curse of that superstition, can live out and out healthier in the heart of a great city than its slaves on the airiest highland of the southern Apeninnes."

Is there not, the reader perhaps asks, danger in the malarious air of the Roman campana or the Charleston meadows ? Yes ! There are regions where air is poisonous. But the only way to escape the poison is not to live in such a region. You might as well allow sewer gas in your bath-room, and expect to escape the poison by keeping the chamber door closed, as to live in a malarious swamp and escape the malaria by trying to live in an air-tight house. Any fresh air is better than any stale air.—*Christian Union.*

